# Platelet transfusions and predictors of bleeding in patients with myelodysplastic syndromes

**DOI:** 10.1111/ejh.14049

**Published:** 2023-07-15

**Authors:** Allison Mo, Erica Wood, Jake Shortt, Erin Hu, Zoe McQuilten

**Affiliations:** ^1^ Transfusion Research Unit, School of Public Health & Preventive Medicine Monash University Melbourne Victoria Australia; ^2^ Monash Haematology Monash Health Melbourne Victoria Australia; ^3^ Austin Pathology and Department of Haematology Austin Health Melbourne Victoria Australia; ^4^ School of Clinical Sciences, Faculty of Medicine, Nursing & Health Sciences Monash University Melbourne Victoria Australia; ^5^ Pharmacy Department Monash Health Melbourne Victoria Australia

**Keywords:** haemorrhage, myelodysplastic syndromes, platelet transfusion, thrombocytopenia

## Abstract

**Objectives:**

This study aimed to describe the burden of thrombocytopenia, supportive care practices, bleeding complications and predictors of bleeding in MDS patients within a large Australian hospital network, to better understand the use and effectiveness of platelet transfusions in MDS.

**Methods:**

A retrospective cohort study of patients aged ≥18 years with MDS, chronic myelomonocytic leukaemia or MDS/myeloproliferative overlap neoplasm admitted from 2016 to 2018 was conducted. Data were obtained from hospital medical records.

**Results:**

One hundred seventy‐nine patients (median age 78 years, 61.5% male) were identified. The median platelet count at first admission was 90 × 10^9^/L. Twenty‐eight (15.6%) patients had severe thrombocytopenia (platelet count <20 × 10^9^/L), of whom nine (32.1%) received prophylactic platelet transfusions, five (17.9%) received tranexamic acid (TXA), seven (25%) received both platelet transfusions and TXA, and seven (25%) received no treatment. Bleeding events requiring hospitalisation occurred in 20 (11.2%) patients. Bleeding was not predicted by presenting platelet count, TXA use, platelet transfusion or anticoagulant/antiplatelet therapies. Three patients died of bleeding, at varying platelet counts (18, 38 and 153 × 10^9^/L).

**Conclusion:**

Thrombocytopenia is common in MDS. Although guidelines recommend otherwise, prophylactic platelet transfusions were commonly used for severe thrombocytopenia. Despite the majority of patients receiving platelet transfusions and/or TXA, 11% developed major bleeding occurring at a wide range of platelet counts.

**Novelty statements**

**What is the new aspect of your work?**

In an area where there are limited existing data and current clinical uncertainty, our cohort study describes treatments and predictors of bleeding in patients with myelodysplastic syndrome (MDS)‐related thrombocytopenia, including the use and effectiveness of platelet transfusions, which are still commonly used despite guidelines recommending otherwise.

**What is the central finding of your work?**

Although evidence‐based guidelines recommend otherwise, we found that prophylactic platelet transfusions were commonly used in MDS patients with severe thrombocytopenia and, despite the majority of patients receiving platelet transfusions and/or tranexamic acid, 11% still developed major bleeding, which occurred at a wide range of platelet counts.

**What is (or could be) the specific clinical relevance of your work?**

Our findings highlight the uncertainty of the benefit of platelet transfusions and tranexamic acid to treat MDS‐related thrombocytopenia, and the vital importance of future clinical trials in this area.

## 1 INTRODUCTION

Thrombocytopenia (platelet count of <100 × 10^9^/L) is common in patients with myelodysplastic syndromes (MDS), affecting 40%–65% of patients.^1^ The incidence of bleeding complications in MDS patients ranges from 3% to 53%.^1^ Platelet transfusions are commonly used in this population, and may be administered as prophylaxis or treatment for bleeding.^2^, ^3^ However, there is limited clinical evidence to guide prophylactic platelet transfusion practice in MDS, which is reflected in the lack of specific recommendations for platelet transfusions in many guidelines.^4^, ^5^ A 2018 Cochrane review comparing therapeutic‐only versus prophylactic platelet transfusion in patients with bone marrow failure disorders identified only one randomised controlled trial which was terminated due to poor recruitment and without reported results.^6^

Platelet transfusions are associated with potential complications, including human leucocyte antigen (HLA) alloimmunisation with the potential to develop platelet refractoriness, allergic and febrile non‐haemolytic reactions and uncommon but potentially very serious consequences such as bacterial contamination.^7^, ^8^ In addition to the hazards associated with transfusion, there is significant economic cost.^2^ Furthermore, the time taken to attend hospital for transfusion and associated tests may interfere with daily activities and be burdensome to patients and families. Access to fresh platelets may also be restricted or not available in more regional or remote locations.

Although there has been recent interest in newer therapeutic agents such as trials of thrombopoiesis‐stimulating agents to treat MDS‐related thrombocytopenia, these are not routinely recommended in clinical practice^9^ and have been associated with inferior clinical outcomes in higher risk MDS patients.^10^ Platelet transfusions remain a cornerstone of supportive care for many patients.

Guidelines generally advise against long‐term prophylactic platelet transfusions in MDS patients with chronic thrombocytopenia, due to the potential risk of complications and the uncertainty of benefit.^4^, ^5^, ^9^ Clinical practice, however, varies widely. An observational study of prophylactic platelet transfusion in haematology patients, including MDS, found only 60% of prophylactic platelet transfusions aligned with clinical guidelines.^11^ Moreover, the optimal management of thrombocytopenic MDS patients with additional risk factors for bleeding is unclear, as highlighted by a recent systematic review.^12^

To better understand the use and effectiveness of platelet transfusions in MDS, we conducted a retrospective cohort study of MDS outpatients at the largest public hospital network in the Australian state of Victoria, with total patient catchment ~1.5 million.

We aimed to first describe the frequency of thrombocytopenia and associated supportive care treatments, including the use of platelet transfusion and other adjunctive therapies to prevent bleeding, and second, the incidence of bleeding and associated risk factors in a cohort of MDS outpatients.

## 2 METHODS

### 2.1 Study design and patient population

Following institutional human research ethics committee approval (HREC reference number RES‐17‐0000‐778Q), we conducted a retrospective cohort study of all adult patients aged ≥18 years with a diagnosis of MDS, chronic myelomonocytic leukaemia (CMML), and other MDS/myeloproliferative overlap neoplasms (MDS/MPN) per 2016 World Health Organization criteria^13^ admitted to the Monash Health hospitals network, in Melbourne, Australia, from 1st August 2016 to 31st July 2018. This tertiary‐level public hospital network includes five adult public hospitals and serves approximately one‐quarter of Melbourne's population, with more than 4 million patient admission episodes per year. Exclusion criteria included a bone marrow blast count of over 20%.

Patients were identified through hospitalisation records. In Australia, all clinical diagnoses and procedures during hospitalisations are recorded using the International Statistical Classification of Diseases and Related Health Problems, Tenth Revision (ICD‐10‐AM),^14^ based on information documented in patients' discharge summaries. We retrieved all hospitalisation records from the Monash Health network during the study period with ICD‐10‐AM codes for MDS (D46.6, D46.7, D46.9, D47.1), CMML (C93.10 and C93.11) and MDS/MPN overlap (C94.70 and C94.71). To mitigate the risk of misclassification due to transcription/transcribing coding errors, we additionally reviewed laboratory and hospital records of all included patients to confirm the MDS/CMML diagnosis.

### 2.2 Data collection

All hospital admission episodes following diagnosis of MDS/CMML were extracted, including demographics, diagnoses and procedural codes for platelet transfusion (ICD‐10‐AM code 1370603) and red cell transfusion (ICD‐10‐AM code 1370602).

Baseline Charlson Comorbidity Index (CCI) was calculated based on ICD‐10‐AM comorbidity codes using a previously validated model.^15^ Haemoglobin, platelet counts, number and type of platelet units transfused and bone marrow biopsy results were obtained from electronic pathology records. Haemoglobin and platelet count on the first day of hospital admission (or on the admission day for day admission episodes) were recorded, for all included admission episodes.

Tranexamic acid (TXA), 5‐azacitidine (AZA; the only hypomethylating agent that is reimbursed under the Australian Pharmaceutical Benefits Scheme [PBS]), antiplatelet and anticoagulant medication prescriptions were obtained from pharmacy dispensing records. Antiplatelet medications which were available on the PBS during this study included aspirin, clopidogrel, prasugrel, ticagrelor, dipyridamole, eptifibatide and tirofiban. Anticoagulants included apixaban, rivaroxaban, fondaparinux, bivalirudin, dabigatran, danaparoid, heparin, enoxaparin, dalteparin, nadroparin and warfarin.

### 2.3 Management of thrombocytopenia

Prophylactic platelet transfusions or TXA for MDS patients were administered at the discretion of the treating clinician. Our hospital policies and procedures reference national patient blood management guidelines, which do not give a specific threshold for platelet transfusion in this setting, and recommend against long‐term prophylactic platelet transfusions in patients with chronic hypoproliferative thrombocytopenia.^4^

All platelet products issued by the national blood service (Australian Red Cross Lifeblood) are leucodepleted and irradiated at source. Type of platelet product is requested by the treating clinician and hospital blood bank and includes pooled platelets (the most commonly administered), apheresis platelets, and, by arrangement, HLA‐matched platelets for patients with platelet refractoriness due to HLA‐alloimmunisation.

### 2.4 Definition of prophylactic platelet transfusion episode

Platelet transfusion may occur during a day admission (hospital admission <24 h duration), or as part of a multi‐day inpatient admission. In this study, we focused on outpatient transfusion episodes only, defined as platelet transfusions administered during day admissions. These transfusions are typically planned in stable outpatients, and are usually prophylactic in nature. This is in comparison with multi‐day admissions where other intercurrent illnesses such as infection or active bleeding are likely to affect platelet transfusion characteristics and platelet counts.

### 2.5 Definition of bleeding events, infections and transfusion reactions

Bleeding events, infections and transfusion reactions were identified by ICD‐10‐AM diagnostic codes (Table S1). As the ICD‐10‐AM codes are based on hospital admissions data, this would only include events which required hospitalisation, or occurred during hospitalisation.

### 2.6 Statistical analysis

Descriptive statistics were used to describe patient characteristics. Risk factors for bleeding and death were identified using logistic regression analysis. For platelet count as a risk factor for bleeding events, we calculated the median platelet count on the first presentation of all hospital admissions prior to the bleeding event.

Comparisons for significance of differences were performed using Chi‐square or Mann–Whitney tests. *p* < .05 was considered statistically significant. Statistical analyses were performed using Stata version 11.0 (StataCorp LP, College Station, TX). Patients were censored at haematopoietic stem cell transplantation (HSCT) or acute myeloid leukaemia (AML) transformation (≥20% blasts), as these events are likely to affect transfusion requirements.

## 3 RESULTS

**FIGURE 1:**
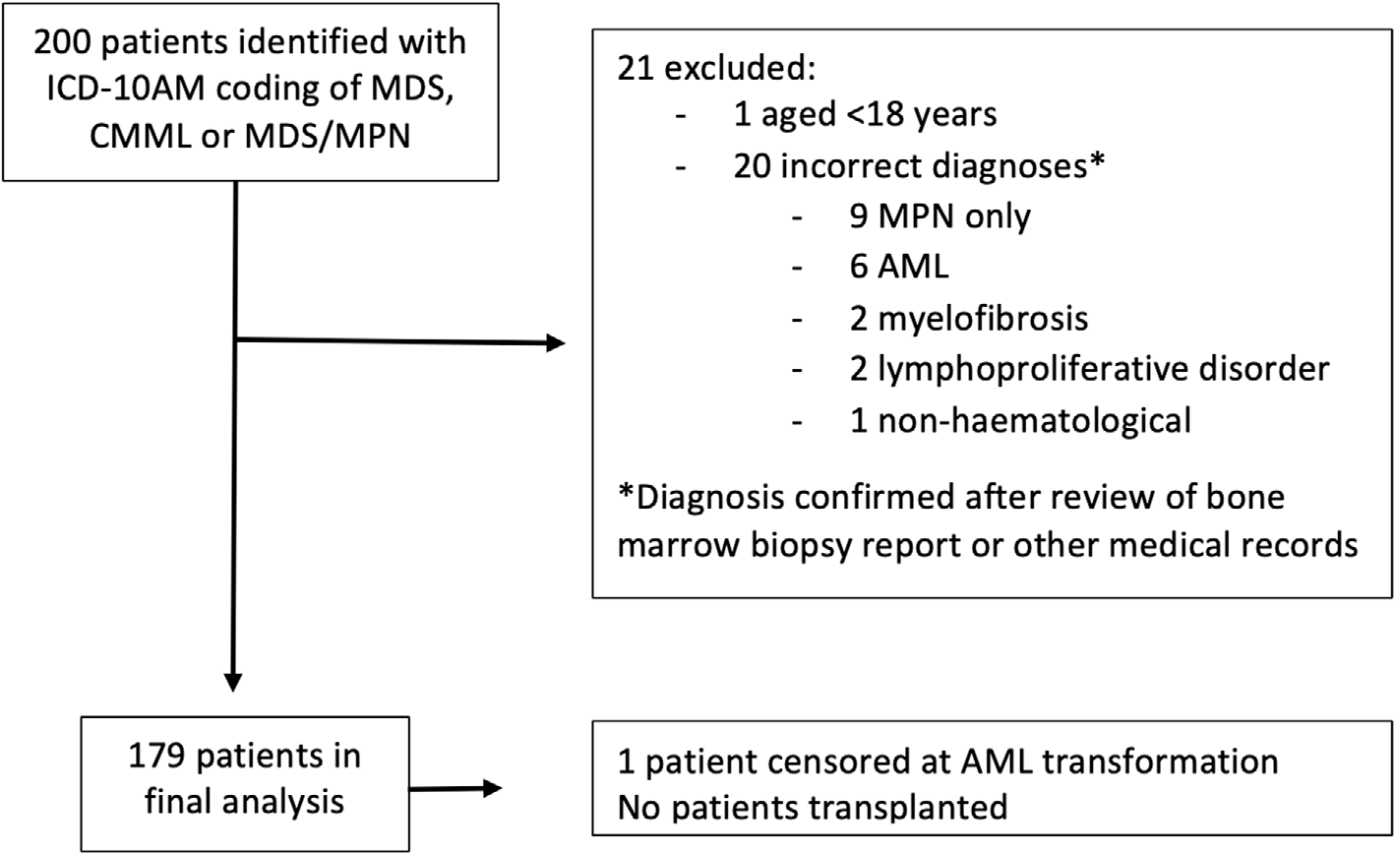
Consort diagram indicating selection of patients included in final analysis. AML, acute myeloid leukaemia; CMML, chronic myelomonocytic leukaemia; ICD‐10 AM, The International Statistical Classification of Diseases and Related Health Problems, Tenth Revision, Australian Modification; MDS, myelodysplastic syndromes; MDS/MPN, MDS/myeloproliferative neoplasm overlap; MPN, myeloproliferative neoplasm.

From August 2016 to July 2018, 200 patients were identified, of whom 21 were excluded from the analysis; 20 for incorrect diagnoses (i.e., not MDS) and one patient as age <18 years (Figure 1). There were 179 patients included in the analysis, who had a total of 804 hospital admissions, including both multi‐day and day admissions.

### 3.1 Patient characteristics

Clinical characteristics are shown in Table 1. Median age was 78 years (IQR 71–84), 110 (61.5%) were male, and 148 (82.7%) had a CCI ≤2 indicating a low burden of comorbidities. The most common WHO MDS classification was MDS with multilineage dysplasia (22.3%). Forty‐eight patients (26.8%) had unknown MDS classification, due to the full bone marrow biopsy results not being available. R‐IPSS risk categories^16^ were: very low (21, 11.7%), low (50, 27.9%), intermediate (15, 8.4%), high (9, 5.0%), very high (9, 5.0%) and unknown in 75 (41.9%).

**TABLE 1:** Clinical characteristics.

Characteristic	*N* = 179
Age in years, median (IQR)	78 (71–84)
Male, *n* (%)	110 (61.5%)
Female, *n* (%)	69 (38.5%)
Charlson comorbidity index (CCI)	
Score 0–2, *n* (%)	148 (82.7%)
Score 3–5, *n* (%)	26 (14.5%)
Score 6+, *n* (%)	5 (2.8%)
MDS classification, *n* (%)	
MDS‐SLD	1 (0.6%)
MDS‐RS‐SLD	5 (2.8%)
MDS‐RS‐MLD	13 (7.3%)
MDS‐MLD	40 (22.3%)
MDS‐EB1	7 (3.9%)
MDS‐EB2	19 (10.6%)
del‐5q	1 (0.6%)
CMML‐0	8 (4.5%)
CMML‐1	11 (6.2%)
CMML‐2	9 (5.0%)
Other MDS/MPN overlap	12 (6.7%)
Therapy‐related MDS	5 (2.8%)
Unknown (bone marrow biopsy not available)	48 (26.8%)
IPSS‐R category, *n* (%)^a^	
Very low risk	21 (11.7%)
Low	50 (27.9%)
Intermediate	15 (8.4%)
High	9 (5.0%)
Very high	9 (5.0%)
Unknown	75 (41.9%)
Platelet count (×10^9^/L), median (IQR) at first presentation to our institution with a diagnosis of MDS	90 (40–146)
Platelet count <50 × 10^9^/L at first presentation to our institution with a diagnosis of MDS, n(%)	35 (19.6%)
Haemoglobin (g/L), median (IQR)	86 (78–95)
AZA, *n* (%)	42 (23.5%)
TXA, *n* (%)	16 (8.9%)
Received antiplatelet or anticoagulant medication	88 (49.2%)
Antiplatelet medication	51 (28.5%)
Anticoagulant medication	22 (12.3%)
Antiplatelet and anticoagulant (in sequential order)	6 (3.4%)
Antiplatelet and anticoagulant (concurrently)	9 (5.0%)
Follow‐up time, weeks, median (IQR)	46 (21–75)
Number of day admissions, median (IQR)	1 (0–3)
Number of multi‐day admissions, median (IQR)	1 (0–2)

^a^ IPSS‐R category unknown due to failed cytogenetics or the diagnostic bone marrow biopsy report not being available.

Abbreviations: AZA, 5‐azacytidine; CMML, chronic myelomonocytic leukaemia; Del‐5q, MDS with isolated del(5q); IPSS‐R, Revised International Prognostic Scoring System; IQR, interquartile range; MDS/MPN overlap, MDS/myeloproliferative neoplasm overlap; MDS‐EB, MDS with excess blasts; MDS‐MLD, MDS with multilineage dysplasia; MDS‐RS‐MLD, MDS with ring sideroblasts and multilineage dysplasia; MDS‐RS‐SLD, MDS with ring sideroblasts and single lineage dysplasia; MDS‐SLD, MDS with single lineage dysplasia; TXA, tranexamic acid.

AZA was given to 42 (23.5%) and TXA to 16 (8.9%) patients. Eighty‐eight (49.2%) of patients received an antiplatelet or anticoagulant medication during the follow‐up period. Fifty‐one (28.5%) received an antiplatelet medication, 22 (12.3%) received an anticoagulant medication, 15 (8.4%) received both an antiplatelet and anticoagulant medication; this included nine patients (5%) receiving these concurrently, and six (3.4%) receiving them sequentially. Of the 35 patients with a platelet count <50 × 10^9^/L at first presentation to our institution, they were less likely to receive antiplatelet/anticoagulant medications during the study period (25.7% vs. 74.3%, *p* = .002).

The median number of day admissions per patient was 1 (IQR 0–3) and multi‐day admissions was 1 (IQR 0–2). Median follow‐up time was 46 weeks (IQR 21–75 weeks).

### 3.2 Thrombocytopenia and treatments

The median platelet count at the first hospital admission in which a diagnosis of MDS was documented was 90 × 10^9^/L (IQR 40–146). The median lowest presenting platelet count across all hospital admissions was 72 × 10^9^/L (IQR 38–142 × 10^9^/L). The lowest presenting platelet count across all hospital admissions was <50 × 10^9^/L in 33.5%, <20 × 10^9^/L in 15.6% and <10 × 10^9^/L in 5.6% of patients (Table 2).

**TABLE 2:** Lowest presenting platelet counts and treatment.

Lowest presenting platelet count (×10^9^/L) (across all hospital admissions)	Number of patients, *n* (%)	Patients transfused OP platelets, *n* (%)	Patients given TXA only, *n* (%)	Patients given TXA and OP platelets, *n* (%)	Patients not given TXA or OP platelets, *n* (%)
All patients	179 (100)	10 (5.6)	9 (5)	7 (3.9)	153 (85.5)
<50	60 (33.5)	9 (15)	8 (13.3)	7 (11.7)	36 (60)
<20	28 (15.6)	9 (32.1)	5 (17.9)	7 (25)	7 (25)
<10	10 (5.6)	3 (30)	3 (30)	4 (40)	0

Abbreviations: OP, outpatient; TXA, tranexamic acid.

Overall, 153 (85.5%) patients did not require treatment for thrombocytopenia. Ten patients (5.6%) received outpatient platelet transfusion alone, nine (5%) received TXA alone and seven (3.9%) received both outpatient platelet transfusion and TXA (Table 2).

Supportive care treatments given for thrombocytopenia are shown in Table 2. For patients with a presenting platelet count across all hospital admissions of <50 × 10^9^/L, 24/60 (40%) were given TXA, outpatient platelet transfusion, or both. However, this increased to 21/28 (75%) for patients with presenting platelet count <20 × 10^9^/L and 10/10 (100%) for presenting platelet count <10 × 10^9^/L. For patients with a platelet count <10 × 10^9^/L, three (30%) received outpatient platelet transfusion, three (30%) received TXA and four (40%) received both TXA and platelet transfusion.

### 3.3 Outpatient platelet transfusions

During the 24‐month study period, 17 patients (9.4%) received outpatient platelet transfusion. There were 87 outpatient platelet transfusion episodes, using a total of 106 therapeutic doses of platelets (one pool of platelets or one apheresis unit). Of all‐day admission episodes, 16.7% (87/521) involved a platelet transfusion.

The median platelet transfusion interval was 7 days (IQR 4–8 days), with a median pre‐transfusion platelet count of 13 × 10^9^/L. Fifty‐one (58.6%) platelet transfusions were administered when the pre‐transfusion platelet count was more than 10 × 10^9^/L. In 10 episodes, a repeat platelet count was measured within 24 h, with a median platelet increment of +12 × 10^9^/L (Table 3).

**TABLE 3:** Characteristics of outpatient platelet transfusions.

Interval (days) between transfusion episodes, median (IQR)	7 (4–8)
No. platelet doses transfused per episode, median (IQR)	1 (1–1)
Types of platelets transfused	
Pooled platelets	63 transfusions (72.4%)
Single‐donor apheresis platelets	24 transfusions (27.6%)
Pre‐transfusion platelet count (×10^9^/L), median (IQR)	13 (11–14)
Number of (%) of times pre‐transfusion platelet count >10 × 10^9^/L	51 (58.6%)
Number of patients with a platelet count taken within 24 h following outpatient platelet transfusion	10
Median platelet increment within 24 h (×10^9^/L), median (IQR) (*n* = 10 patients)	+12 (+2 to +17)

Abbreviation: IQR, interquartile range.

In 68 (78.25%) admissions, one adult therapeutic dose of platelets was transfused. Nineteen admissions (21.9%) involved two doses, at similar pre‐transfusion platelet counts (one unit: median 11 × 10^9^/L, IQR 9–16 vs. two units: median 11.5 × 10^9^/L, IQR 7–13, *p* = .38).

Twenty‐four transfusions (27.6%) involved apheresis platelets, including three patients requiring HLA‐matched platelets. In the 10 patients with a platelet count taken within 24 h, there was no significant difference in post‐transfusion platelet increment between different platelet product types (median [IQR] change in platelet count for pooled platelets was +12 × 10^9^/L [5–17 × 10^9^/L] vs. single donor apheresis platelets +18 × 10^9^/ [IQR 2–53 × 10^9^/L]; *p* = .69) or different number of doses transfused (median [IQR] change in platelet count for one platelet unit +13 × 10^9^/L vs. two platelet units +2 [−4 to –35]; *p* = .49).

### 3.4 Bleeding events

Over a median follow‐up time of 46 weeks, bleeding events requiring or occurring during hospitalisation occurred in 20 (11.2%) patients, with no significant difference between platelet‐transfused patients (6/34, 17.7%) and non‐transfused patients (14/145, 9.7%, *p* = .18). Twenty patients experienced 25 total bleeding events: 13 episodes of gastrointestinal bleeding, one intracranial haemorrhage and 11 bleeding events at other sites (four respiratory tract, three urinary tract, one had both renal tract and respiratory tract bleeding, three unspecified). Three patients died of bleeding: one fatal intracranial haemorrhage and two gastrointestinal haemorrhages, with presenting platelet counts of 18, 38 and 153 × 10^9^/L.

Neither presenting platelet count, IPSS‐R score, prior TXA use, prior platelet transfusion nor antiplatelet/anticoagulant use was associated with increased risk of first bleeding event (Table 4).

**TABLE 4:** Predictive factors of first bleeding event.

	Bleeding (*n* = 20 patients)	No bleeding (*n* = 159 patients)	*p*‐value
Platelet count (×10^9^/L), median (IQR)	67 (4–150)	98 (55–184)	.57
TXA use prior to first bleeding event, *n* (%)	3 (15.0%)	13 (8.2%)	.31
Platelet transfusion prior to first bleeding event, *n* (%)	6 (30.0%)	28 (17.6%)	.18
Red cell transfusion dependence (defined as 2 or more red cell transfusions within 16 weeks)	8 (40%)	38 (23.9%)	.120
Infection prior to or during current admission, *n* (%)	13 (65.0%)	39 (24.5%)	**<.001**
Age (years) at first hospital admission, median (IQR)	78.5 (73.5–83.5)	78.0 (71.0–84.0)	.58
AZA use, *n* (%)	6 (30%)	36 (22.6%)	.46
Anticoagulant or antiplatelet medication use (at time of first bleeding event; or at any time for those without bleeding), *n* (%)	10 (50%)	78 (49.1%)	.40
Antiplatelet medication	6 (30%)	46 (28.9%)	
Anticoagulant medication	3 (15%)	22 (13.8%)	
Antiplatelet and anticoagulant, concurrently	1 (5%)	6 (3.8%)	
Antiplatelet and anticoagulation, sequentially	–	4 (2.5%)	
Gender, male, *n* (%)	16 (80.0%)	93 (59.2%)	.07
CCI (median, IQR)	1 (0–2)	0 (0–2)	.08
Renal comorbidity, *n* (%)	4 (20.0%)	9 (5.7%)	**.02**
MDS classification, *n* (%)			.34
MDS‐SLD	0	1 (0.6%)	
MDS‐RS‐SLD	0	5 (3.1%)	
MDS‐RS‐MLD	0	13 (8.2%)	
MDS‐MLD	3 (15.0%)	37 (23.3%)	
MDS‐EB1	1 (5.0%)	6 (3.8%)	
MDS‐EB2	2 (10.0%)	17 (10.7%)	
del‐5q	0	1 (0.6%)	
CMML‐0	0	8 (5.0%)	
CMML‐1	1 (5.0%)	10 (6.3%)	
CMML‐2	3 (15.0%)	6 (3.8%)	
Other MDS/MPN overlap	3 (15.0%)	9 (5.7%)	
Therapy‐related MDS	0	5 (3.1%)	
Unknown (bone marrow biopsy not available)	7 (35.0%)	41 (25.8%)	
IPSS‐R category^a^, *n* (%)			.66
Total with known IPSS‐R category	11	93	
Very low	1/11 (9.1%)	20/93 (21.5%)	
Low	6/11 (54.6%)	44/93 (47.3%)	
Intermediate	1/11 (9.1%)	14/93 (15.1%)	
High	2/11 (18.2%)	7/93 (7.5%)	
Very high	1/11 (9.1%)	8/93 (8.6%)	

^a^ IPSS‐R category known in 104 patients only. The remainder are unknown due to failed cytogenetics or the diagnostic bone marrow biopsy report not being available.

Abbreviations: AZA, azacitidine; CCI, Charlson Comorbidity Index score; CMML, chronic myelomonocytic leukaemia; Del‐5q, MDS with isolated del(5q); IQR, interquartile range; MDS/MPN overlap, MDS/myeloproliferative neoplasm overlap; MDS‐EB, MDS with excess blasts; MDS‐MLD, MDS with multilineage dysplasia; MDS‐RS‐MLD, MDS with ring sideroblasts and multilineage dysplasia; MDS‐RS‐SLD, MDS with ring sideroblasts and single lineage dysplasia; MDS‐SLD, MDS with single lineage dysplasia; TXA, tranexamic acid.

Of the 35 patients with a first presenting platelet count of less than 50 × 10^9^/L at study entry, nine patients (25.8%) received antiplatelet/anticoagulant medications during the follow‐up period and none had a bleeding episode; in comparison, bleeding occurred in 5/26 patients (19.2%) who did not receive antiplatelet/anticoagulant medication.

Infection during a previous or current admission (65.0% vs. 24.5%, *p* < .001) and comorbid renal disease (20% vs. 5.7%, *p* = .02) were associated with first bleeding event (Table 4).

### 3.5 Transfusion reactions

No transfusion reactions were recorded in the discharge summary using ICD‐10‐AM codes.

## 4 DISCUSSION

This observational cohort study from a large multi‐campus tertiary health service demonstrates that thrombocytopenia is common in MDS patients, that bleeding affects approximately one in 10 patients, and that prophylactic outpatient platelet transfusions are commonly administered in real‐world practice, despite the lack of evidence and contrary to current guidelines.^4^, ^5^ Further, despite the majority of patients receiving platelet transfusion and/or TXA, 11% developed major bleeding, occurring at a wide range of platelet counts and irrespective of antiplatelet/anticoagulant use.

There are few studies describing real‐world use of platelet transfusions and TXA in MDS patients. A single‐centre Canadian retrospective audit of 586 MDS patients found that 99 patients (17%) had severe persistent thrombocytopenia (platelet count <20 × 10^9^/L) and, despite disparities in clinical treatment with 58% eventually receiving prophylactic platelet transfusions, 12% receiving only therapeutic platelet transfusions, 19% receiving only TXA, and 10% requiring no treatment for their thrombocytopenia, there was no significant difference in grades 3–4 bleeding rates between the groups.^17^

We similarly found no association between thrombocytopenia treatment and bleeding events. In this study, 11% of patients experienced bleeding, including three fatal events. Although we did not grade the bleeding events, as our study only captured in‐hospital bleeding events, these would generally be of more serious nature. Of note, bleeding risk was not related to presenting platelet count, nor prior platelet transfusion or TXA use. Further, the use of antiplatelet/anticoagulant medications was common in this cohort (49.2%) but was not associated with bleeding events, including in patients with a presenting platelet count of less than 50 × 10^9^/L. Although the numbers are small, the three patients who died of bleeding (one intracranial haemorrhage and two gastrointestinal haemorrhages) had wide variations in their presenting platelet counts (18, 38 and 153 × 10^9^/L).

Infection was identified as a potential risk factors for bleeding in our study. Fever can increase bleeding risk in patients with AML,^18^ and infection may also lead to organ dysfunction, coagulopathy, localised bleeding at the site of infection or use of antibiotics that have an antiplatelet effect. Inflammation has been associated with increased bleeding risk in other patient populations, such as patients with atrial fibrillation,^19^ after acute coronary syndromes^20^ or exacerbating intracerebral haemorrhage‐induced brain injury.^21^

Together, these observations raise the question of whether factors beyond the platelet count alone in MDS patients predict bleeding and indicate that further prospective clinical trials, together with biomarker studies, are warranted to determine the efficacy of TXA, prophylactic platelet transfusion and other interventions to prevent bleeding in MDS patients. Platelet dysfunction, for example, has been reported in MDS patients^22^ and may account for differential bleeding risks in patients, independent of the platelet count or the use of antiplatelet/anticoagulant medications. Further, though IPSS‐R score did not predict bleeding risk in this cohort, we did not have data on gene mutation profiling due to the study period being prior to routine use of next‐generation sequencing. Gene mutation profiling of patients with intracerebral haemorrhage has identified biomarkers associated with haemorrhage progression; however, these have not been explored in MDS‐related bleeding.^23^

Our study, similar to the Canadian audit,^17^ demonstrated there is variation in clinical practice for patients with severe thrombocytopenia (platelet count <20 × 10^9^/L); in our cohort, 32% received outpatient platelet transfusions alone, 25% received both platelet transfusions and TXA, 18% received TXA alone and 25% not on treatment. A recent Dutch clinical practice survey similarly found clinical practice variation; for MDS patients on hypomethylating agents, 87% of Dutch haematologists would prescribe prophylactic platelet transfusions and 80% would prescribe TXA.^24^ For MDS patients not on active treatment, 35% said they would prescribe prophylactic platelet transfusions and 75% prescribe TXA, with TXA used mainly for patients with a tendency to have bleeding.^24^

Although this study evaluated fewer patients than the Canadian study, we also included detailed data on individual transfusion requirements and pre‐ and post‐transfusion blood counts. We found a high burden of transfusion in those who received outpatient platelet transfusion with a median transfusion interval of 7 days, and 22% involved two adult platelet doses. Approximately 28% of platelet transfusions were apheresis units, including three patients requiring HLA‐matched platelets. These products are more costly and resource‐intensive to acquire; in Australia, the unit price for one dose of apheresis single‐donor platelet is 531 AUD compared to 244 AUD for pooled platelets.^25^

The median pre‐transfusion platelet count in our outpatient cohort was 13 × 10^9^/L. Guidelines provide no recommendation on platelet transfusion threshold in MDS patients; however, clinicians may have extrapolated from other clinical settings, such as patients with acute leukaemia or HSCT, whereby a prophylactic platelet transfusion threshold of 10 × 10^9^ is recommended.^4^, ^26^, ^27^ Unlike in MDS, such patients have a temporary period of thrombocytopenia with expected bone marrow recovery. Few studies of platelet thresholds to reduce bleeding exist in chronic hypoproliferative thrombocytopenia. One small study of 22 untransfused aplastic anaemia patients demonstrated that faecal blood loss did not substantially increase until platelet counts were 5 × 10^9^/L or less.^28^

Compared to the Canadian observational study and the Dutch survey findings, a relatively smaller percentage of patients in our study (9%) were prescribed TXA. However, this proportion increased to 70% of patients with a nadir platelet count <10 × 10^9^/L. TXA is an inexpensive antifibrinolytic agent which reduces bleeding in patients with trauma,^29^ surgery^30^ and postpartum haemorrhage.^31^ There are no guidelines on TXA use in hypoproliferative thrombocytopenia. The recently published American Trial using Tranexamic Acid in Thrombocytopenia (A‐TREAT) study demonstrated no difference in bleeding outcomes in haematology patients with chemotherapy‐induced thrombocytopenia randomized to receive TXA prophylaxis versus placebo.^32^ The results of a similar trial in UK and Australia are awaited.^33^ However, studies in MDS outpatients with chronic thrombocytopenia, a different patient population to that in A‐TREAT, are lacking. In their retrospective study, Vijenthira et al. showed no significant differences in grade 3–4 bleeding rates between severely thrombocytopenic MDS patients treated with prophylactic platelet transfusions, TXA or no treatment.^17^ We similarly found no association between TXA or prior platelet transfusion, and bleeding events. In all, these observations indicate that further prospective clinical trials are warranted to determine the efficacy of TXA, prophylactic platelet transfusion and other interventions to prevent bleeding in MDS patients.

Notably, no transfusion reactions were documented in our study. This may reflect under‐recognition and under‐reporting of events in an outpatient transfusion setting. Coding may also be problematic due to a lack of ICD‐10‐AM codes for a number of transfusion reactions. There are only five ICD‐10‐AM codes relating to transfusion reactions: ABO incompatibility, Rh incompatibility, anaphylactic shock due to serum, other serum reaction, shock during or result from a procedure not otherwise specified (see Table S1).

This study has several limitations, including the retrospective observational design, and focuses on an Australian context. We were not able to determine the clinical reasons for instituting prophylactic platelet transfusion or TXA in our cohort. IPSS‐R category was unknown in 41% due to failed cytogenetics or the full bone marrow biopsy report being unavailable. Use of the hospital ICD‐10‐AM data has limitations, including coding or transcription errors. We were not able to capture events experienced in the community. Although these are likely to be less severe, minor bleeding events may still be distressing, affect patients' quality of life, or impact on clinical decisions about prophylactic or therapeutic treatments for thrombocytopenia. Future prospective studies should focus on capturing such episodes in an outpatient setting.

Strengths of this study include real‐world data in an area that has previously not been well described, including detailed data on transfusion rates, frequencies, blood product use and bleeding outcomes.

## 5 CONCLUSIONS

In conclusion, this study confirms that, in a real‐world setting, thrombocytopenia is common and platelet transfusions are commonly administered for the management of MDS patients, despite the lack of data for their efficacy in this setting and our own findings suggesting that other clinical factors, beyond the presenting platelet count alone, may be associated with bleeding outcomes. Our study highlights the need for clinical trials to determine the predictors of bleeding, and the efficacy and safety of platelet transfusions, TXA or other therapeutic agents, in MDS patients. Future trials should focus on outcomes important to patients, such as clinically significant bleeding and quality of life, not just laboratory results such as platelet counts. They should also incorporate health economics outcomes, given the costs and other resources required for administering regular platelet transfusions, and the potential burden for patients, including frequent hospital admission.

## AUTHOR CONTRIBUTIONS

Allison Mo, Zoe McQuilten, Erica Wood and Jake Shortt developed the study concept and design. Allison Mo and Erin Hu performed the data collection. Allison Mo and Zoe McQuilten conducted the data analysis. Allison Mo, Zoe McQuilten, Erica Wood, Jake Shortt and Erin Hu wrote and edited the manuscript.

## FUNDING INFORMATION

Allison Mo receives PhD scholarship funding from the National Health and Medical Research Council (NHMRC), National Blood Authority, Haematology Society of Australia and New Zealand and Monash University. Jake Shortt and Zoe McQuilten are supported by an Australian NHMRC Emerging Leadership Fellowships. Erica Wood is supported by NHMRC Leadership Fellowship.

## CONFLICT OF INTEREST STATEMENT

The authors do not have any conflict of interest to disclose for the submitted work. Jake Shortt has served on advisory boards for Astellas, Bristol Myers Squibb, Otsuka, Mundipharma and Novartis, received research funding from Amgen, Astex and Bristol Myers Squibb and received speaker's fees from Mundipharma (all disclosures for Jake Shortt are outside of the submitted work).

## Supporting information


**Table S1:** ICD‐10‐AM diagnostic codes.

## Data Availability

The authors confirm that the data that support the findings of this study are available on request from the corresponding author. The data are not publicly available due to privacy and ethical restrictions.
