# Supplementary material for: Platelet transfusions and predictors of bleeding in patients with myelodysplastic syndromes
Source: Eur J Haematol. 2023 Jul 15;111(4):592–600. doi: 10.1111/ejh.14049 (PMC10952506; doi:10.1111/ejh.14049)
Supplement: Supplementary file 1 — Table S1: ICD‐10‐AM diagnostic codes. [file EJH-111-592-s001.docx]

**Supplementary table 1: ICD-10-AM diagnostic codes**

**BLEEDING**

| **ICD-10-AM code** | **DIAGNOSIS** |
| --- | --- |
| **INTRACRANIAL BLEEDING** | |
| I60 | Subarachnoid haemorrhage |
| I60.0 | Subarachnoid haemorrhage, carotid siphon & bifurcation |
| I60.1 | Subarachnoid haemorrhage from middle cerebral artery |
| I60.2 | Subarachnoid haemorrhage from ant communicating artery |
| I60.3 | Subarachnoid haemorrhage from post comm art artery |
| I60.4 | Subarachnoid haemorrhage from basilar artery |
| I60.5 | Subarachnoid haemorrhage from vertebral art artery |
| I60.6 | Subarachnoid haemorrhage from other intracranial art artery |
| I60.7 | Subarachnoid haemorrhage from intracranial artery unspecified |
| I60.8 | Other Subarachnoid haemorrhage |
| I60.9 | Subarachnoid haemorrhage unspecified |
| I61 | Intracerebral haemorrhage |
| I61.0 | Intracerebral haemorrhage in hemisphere subcortical |
| I61.1 | Intracerebal haemorrhage in hemisphere cortical |
| I61.2 | Intracerebral haemorrhage in hemisphere unspecified |
| I61.3 | Intracerebral haemorrhage in brain stem |
| I61.4 | Intracerebral haemorrhage in cerebellum |
| I61.5 | Intracerebral haemorrhage intraventricular |
| I61.6 | Intracerebral haemorrhage multiple localised |
| I61.8 | Other intracerebral haemorrhage |
| I61.9 | Intracerebral haemorrhage unspecified |
| I62 | Other nontraumatic intracranial haemorrhage |
| I62.0 | Subdural haemorrhage (acute)(nontraumatic) |
| I62.1 | Nontraumatic extradural |
| I62.9 | Intracranial, nontraumatic, unspecified |
| T79.2 | Traumatic Subarachnoid haemorrhage – not included |
| **GASTROINTESTINAL BLEEDING** | |
| K92.0 | Haemetemesis |
| K92.1 | Melena |
| I85.0 | Oesophageal varices with bleeding |
| I98.3 | Oesophageal varices with bleeding in disease classified elsewhere |
| K22.10 | \| Ulcer of oesophagus, acute with bleeding \| \| --- \| |
| K22.12 | \| Ulcer of oesophagus, acute with both bleeding and perforation \| \| --- \| |
| K22.14 | \| Ulcer of oesophagus, chronic or unspecified with bleeding \| \| --- \| |
| K22.16 | \| Ulcer of oesophagus, chronic or unspecified with both bleeding and perforation \| \| --- \| |
| K25.0 | \| Gastric ulcer, acute with bleeding \| \| --- \| |
| K25.2 | \| Gastric ulcer, acute with both bleeding and perforation \| \| --- \| |
| K25.4 | \| Gastric ulcer, chronic or unspecified with bleeding \| \| --- \| |
| K25.6 | \| Gastric ulcer, chronic or unspecified with both bleeding and perforation \| \| --- \| |
| K26.0 | \| Duodenal ulcer, acute with bleeding \| \| --- \| |
| K26.2 | \| Duodenal ulcer, acute with both bleeding and perforation \| \| --- \| |
| K26.4 | \| Duodenal ulcer, chronic or unspecified with bleeding \| \| --- \| |
| K26.6 | \| Duodenal ulcer, chronic or unspecified with both bleeding and perforation \| \| --- \| |
| K27.0 | Peptic ulcer, acute with bleeding |
| K27.2 | Peptic ulcer, acute with both bleeding and perforation |
| K27.4 | Peptic ulcer, chronic or unspecified with bleeding |
| K27.6 | Peptic ulcer, chronic or unspecified with both bleeding and perforation |
| K28.0 | Acute Gastrojejunal ulcer with haemorrhage |
| K28.2 | Acute Gastrojejunal ulcer, acute with haemorrhage and perforation |
| K28.4 | Gastrojejunal ulcer, chronic or unspecified with haemorrhage |
| K28.6 | Gastrojejunal ulcer, chronic or unspecified with haemorrhage and perforation |
| K29.0 | Acute haemorrhagic gastritis |
| K29.2 | Alcoholic gastritis with haemorrhage |
| K29.3 | Chronic superficial gastritis with haemorrhage |
| K29.4 | Chronic atrophic gastritis with haemorrhage |
| K29.5 | Chronic gastritis, unspecified, with haemorrhage |
| K29.6 | Other gastritis with haemorrhage |
| K29.7 | Gastritis unspecified with haemorrhage |
| K29.8 | Duodenitis with haemorrhage |
| K29.9 | Gastroduodenitis unspecified with haemorrhage |
| K31.82 | Angiodysplasia of stomach and duodenum with haemorrhage |
| K55.22 | Angiodysplasia of colon with bleeding |
| K62.5 | Haemorrhage of anus and rectum |
| K92.2 | Gastrointestinal haemorrhage, unspecified |
| K57.01 | Diverticulosis of small intestine with haemorrhage, perforation and abscess |
| K57.03 | Diverticulitis of small intestine with haemorrhage, perforation and abscess |
| K57.11 | Diverticulosis of small intestine without perforation or abscess, with haemorrhage |
| K57.21 | Diverticulosis of large intestine with haemorrhage, perforation and abscess |
| K57.31 | Diverticulosis of large intestine without perforation or abscess, with haemorrhage |
| K57.41 | Diverticulosis of both small and large intestine with haemorrhage, perforation and abscess |
| K57.43 | Diverticulitis of both small and large intestine with haemorrhage, perforation and abscess |
| K57.51 | Diverticulosis of both small and large intestine without perforation or abscess, with haemorrhage |
| K57.53 | Diverticulitis of both small and large intestine without perforation or abscess, with haemorrhage |
| K57.83 | Diverticulitis of intestine, artery unspecified, with haemorrhage, perforation and abscess |
| K57.91 | Diverticulosis of intestine, artery unspecified, without perforation or abscess, with haemorrhage |
| K57.93 | Diverticulitis of intestine, artery unspecified, without perforation or abscess, with haemorrhage |
| K92.2 | Gastrointestinal haemorrhage unspecified |
| **OTHER BLEEDING SITES** | |
| Renal tract bleeding | |
| R31 | Unspecified haematuria |
| N39.81 | Loin pain / haematuria syndrome |
| N02.0 | Recurrent and persistent haematuria, minor glomerular abnormality |
| N02.1 | Recurrent and persistent haematuria, focal and segmental glomerular lesions |
| N02.2 | Recurrent and persistent haematuria, diffuse membranous glomerulonephritis |
| N02.3 | Recurrent and persistent haematuria, diffuse mesangial proliferative glomerulonephritis |
| N02.4 | Recurrent and persistent haematuria, diffuse endocapillary proliferative glomerulonephritis |
| N02.5 | Recurrent and persistent haematuria, diffuse mesangiocapillary glomerulonephritis |
| N02.6 | Recurrent and persistent haematuria, dense deposit disease |
| N02.7 | Recurrent and persistent haematuria, diffuse crescentic glomerulonephritis |
| N02.8 | Recurrent and persistent haematuria, other |
| N02.9 | Recurrent and persistent haematuria, unspecified |
| Prostate bleeding | |
| N42.1 | Congestion and haemorrhage of prostate |
| Haemoperitoneum | |
| K66.1 | Haemoperitoneum |
| Gynaecological bleeding | |
| N92.3 | Ovulation bleeding |
| N92.4 | Excessive bleeding premenopausal period |
| N93 | Other abnormal uterine and vaginal bleeding |
| N93.8 | Other specified abnormal uterine and vaginal bleeding |
| N93.9 | Abnormal uterine and vaginal bleeding, unspecified |
| N95.0 | Postmenopausal bleeding |
| Respiratory tract bleeding | |
| R04 | Haemorrhage from respiratory passages |
| R04.0 | Epistaxis |
| R04.1 | Haemorrhage from throat |
| R04.2 | Haemoptysis |
| R04.8 | Bleeding from other sites in respiratory passages |
| R04.9 | Bleeding from respiratory passages, unspecified |
| Opthalmologic bleeding | |
| H11.3 | Conjunctival haemorrhage |
| H31.3 | Choroidal haemorrhage and rupture |
| H45.0 | Vitreous haemorrhage in diseases classified elsewhere |
| H35.6 | Retinal haemorrhage |
| H43.1 | Vitreous haemorrhage |
| H45.0 | Vitreous bleeding in diseases classified elsewhere |
| Joint bleeding | |
| M25.0 | Haemarthrosis |
| M25.00 | Haemarthrosis multiple sites |
| M25.01 | Haemarthrosis shoulder region |
| M25.02 | Haemarthrosis upper arm |
| M25.03 | Haemarthrosis forearm arm |
| M25.04 | Haemarthrosis hand |
| M25.05 | Haemarthrosis pelvic region and thigh |
| M25.06 | Haemarthrosis lower leg |
| M25.07 | Haemarthrosis ankle and foot |
| M25.08 | Haemarthrosis other |
| M25.09 | Haemarthrosis site unspecified |
| Spinal tract bleeding | |
| S06.4 | Epidural haemorrhage |
| Site not specified | |
| R58 | Haemorrhage not elsewhere classified |
| D69.9 | Haemorrhagic condition, unspecified |
| D69 | Purpura and other haemorrhagic conditions |
| D68.3 | Haemorrhagic disorder due to circulating anticoagulants |

**TRANSFUSION REACTIONS**

| **ICD-10AM CODE** | **DIAGNOSIS** |
| --- | --- |
| T803 | ABO incompatibility reaction |
| T804 | Rh incompatibility reaction |
| T805 | Anaphylactic shock due to serum |
| T806 | Other serum reactions |
| T811 | Shock during or resulting from a procedure, not elsewhere classified |

**INFECTIONS**

| **ICD-10AM CODE** | **DIAGNOSIS** |
| --- | --- |
| **FUNGAL** |  |
| B37 | Candidiasis |
| B37.7 | Candida sepsis |
| B38 | Coccidioidomycosis |
| B39 | Histoplasmosis |
| B40 | Blastomycosis |
| B41 | Paracoccidioidomycosis |
| B42 | Sporotrichosis |
| B43 | Chromomycosis and phaeomycotic abscess |
| B44 | Aspergillosis |
| B45 | Cryptococcosis |
| B46 | Zygomycosis |
| B47 | Mycetoma |
| B48 | Other mycoses, not elsewhere classified |
| B49 | Unspecified mycosis |
| **BACTERIAL** |  |
| A02 | Salmonella infections (excluding typhoid and paratyphoid) |
| A03 | Shigellosis |
| A04 | Other bacterial intestinal infections |
| A15 | Respiratory tuberculosis, bacteriologically and histologically confirmed |
| A16 | Respiratory tuberculosis confirmed bacteriologically or histologically |
| A17 | Tuberculosis of nervous system |
| A18 | Tuberculosis of other organs |
| A19 | Miliary tuberculosis |
| A31 | Infection due to other mycobacteria |
| A32 | Listeriosis |
| A327 | Disseminated listeriosis |
| A38 | Scarlet fever |
| A39 | Meningococcal infection |
| A39.4 | Meningococcocaemia, unspecified |
| A40 | Streptococal sepsis |
| A41 | Other sepsis |
| A41.0 | Sepsis due to *Staphylococcus aureus* |
| A41.1 | Sepsis due to Coagulase-negative staphylococcus |
| A41.2 | Sepsis due to unspecified staphylococcus |
| A41.3 | Sepsis due to *Haemophilus influenza* |
| A41.4 | Sepsis due to anaerobes |
| A41.5 | Gram-negative septicaemia NOS |
| A41.51 | Sepsis due to *Escherichia coli* |
| A41.52 | Sepsis due to Pseudomonas |
| A41.58 | Sepsis due to other Gram-negative organisms |
| A41.8 | Other specified sepsis |
| A41.9 | Sepsis unspecified, septicaemia |
| A42 | Actinomycosis |
| A43 | Norcardiosis |
| A44 | Bartonellosis |
| A46 | Erysipelas |
| A48 | Other bacterial disease, elsewhere classified |
| A48.1 | Legionnaires disease |
| A48.3 | Toxic shock syndrome |
| A49 | Bacterial infection of unspecified site |
| A54 | Gonococcal infection |
| A54.8 | Other gonococcal infection |
| A78 | Q fever |
| B95 | Streptococal and staphylococcal other |
| B96 | Other bacterial |
| G00 | Bacterial meningitis |
| I33 | Acute and subacute endocarditis |
| J13 | Pneumonia due to Streptococcal pneumoniae |
| J14 | Pneumonia due to Haemophilus influenzae |
| J15 | Bacterial pneumonia |
| J16 | Pneumonia due to other infective organisms |
| J17 | Pneumonia |
| J18 | Pneumonia organism unspecified |
| J85 | Abscess of lung and mediastinum |
| K57 | Diverticular disease of intestine |
| K61 | Abscess of anal and rectal regions |
| K65 | Peritonitis |
| L03 | Cellulitis |
| M00 | Pyogenic arthritis |
| M86 | Osteomyelitis |
| **VIRAL** |  |
| A08 | Viral and other intestinal infections |
| A08.0 | Rotaviral enteritis |
| A08.2 | Adenoviral enteritis |
| A08.3 | Other viral enteritis |
| A08.4 | Viral intestinal infection unspecified |
| A60 | Herpes simplex infection |
| A60.0 | Herpes viral infection genitalia and urinary tract |
| A60.1 | Herpes viral infection perianal skin rectum |
| A60.9 | Herpes viral infection unspecified |
| A81.8 | Other atypical viral infection CNS |
| A81.9 | Atypical viral infection CNS unspecified |
| A87 | Viral meningitis |
| A87.0 | Enteroviral meningitis |
| A87.1 | Adenoviral meningitis |
| A87.8 | Other viral meningitis |
| A87.9 | Viral meningitis unspecified |
| A88 | Other viral infections of CNS |
| A89 | Unspecified viral infection of CNS |
| B00 | Herpes viral infection (herpes simplex) |
| B00.2 | Herpes gingival stomatitis pharyngotonsillitis |
| B00.3 | Herpes viral meningitis |
| B00.4 | Herpes viral encephalitis |
| B00.5 | Herpes viral ocular disease |
| B00.7 | Disseminated herpes viral disease |
| B00.8 | Other forms of herpes viral infection |
| B00.9 | Herpes viral infection unspecified |
| B01 | Varicella (chicken pox) |
| B01.0 | Varicella meningitis |
| B01.1 | Varicella encephalitis |
| B01.2 | Varicella pneumonia |
| B01.8 | Varicella with other complications |
| B01.9 | Varicella without complications |
| B02 | Zoster (herpes zoster) |
| B02.0 | Zoster encephalitis |
| B02.1 | Zoster meningitis |
| B02.2 | Zoster with other nervous system involvement |
| B02.3 | Zoster ocular disease |
| B02.7 | Disseminated zoster |
| B02.8 | Zoster with other complications |
| B02.9 | Zoster without complications |
| B05 | Measles |
| B05.0 | Measles complicated by encephalitis |
| B05.1 | Measles complicated by meningitis |
| B05.2 | Measles complicated by pneumonia |
| B05.3 | Measles complicated by otitis media |
| B05.4 | Measles with intestinal complications |
| B05.8 | Measles with other complications |
| B05.9 | Measles without complications |
| B06 | Rubella |
| B06.0 | Rubella with neurological complications |
| B07 | Viral warts |
| B08.8 | Other orthopox virus infection |
| B09 | Unspecified infection of skin and mucosa |
| B15 | Acute hepatitis A |
| B15.0 | Hepatitis A with hepatic coma |
| B15.9 | Hepatitis A without hepatic coma |
| B16 | Acute hepatitis B |
| B16.0 | Acute hepatitis B with delta agent with hepatic coma |
| B16.1 | Acute hepatitis B with delta agent without hepatic coma |
| B16.2 | Acute hepatitis B without delta agent with hepatic coma |
| B16.9 | Acute hepatitis B without delta agent without hepatic coma |
| B17 | Other acute viral hepatitis |
| B17.0 | Acute delta (super) infection in hep B carrier |
| B17.1 | Acute hepatitis C |
| B17.2 | Acute hepatitis E |
| B17.8 | Other specified acute viral hepatitis |
| B18 | Chronic viral hepatitis |
| B18.0 | Chronic hepatitis B with delta agent |
| B18.1 | Chronic hepatitis B without delta agent |
| B18.8 | Other chronic viral hepatitis |
| B18.9 | Chronic viral hepatitis unspecified |
| B19.0 | Unspecified viral hepatitis with hepatic coma |
| B19.9 | Unspecified viral hepatitis without hepatic coma |
| B23.0 | Acute HIV infection syndrome |
| B25 | Cytomegaloviral disease |
| B25.0 | Cytomegaloviral pneumonitis |
| B25.1 | Cytomegaloviral hepatitis |
| B25.2 | Cytomegaloviral pancreatitis |
| B25.8 | Other Cytomegaloviral disease |
| B25.9 | Cytomegaloviral disease unspecified |
| B26 | Mumps |
| B27 | Infectious mononucleosis |
| B27.0 | Gamma herpesviral mononucleosis |
| B27.8 | Other infectious mononucleosis |
| B27.9 | Infectious mononucleosis unspecified |
| B33 | Other viral diseases, not elsewhere classified |
| B33.8 | Other specified viral disease |
| B34.8 | Other viral infections of unspecified site |
| B34.9 | Viral infection unspecified |
| J10 | Influenza other influenza virus |
| J10.0 | Influenza with pneumonia, other virus identified |
| J10.1 | Influenza with other respiratory manifestations, other virus identified |
| J10.8 | Influenza with other manifestation, other virus identified |
| J11 | Influenza virus not identified |
| J11.0 | Influenza with pneumonia, virus not identified |
| J11.1 | Influenza with other respiratory manifestation, virus not identified |
| J11.8 | Influenza with other manifestation, virus not identified |
| J12 | Viral pneumonia not elsewhere classified |
| J12.3 | Human metapneumovirus pneumonia |
| J12.8 | Other viral pneumonia |
| J12.9 | Viral pneumonia unspecified |
